# Acute Generalized Exanthematous Pustulosis After COVID‐19 Infection: A Case Report From Saudi Arabia

**DOI:** 10.7759/cureus.11609

**Published:** 2020-11-21

**Authors:** Malak J Alzahrani, Mohamed M Moussa, Dunya Alfaraj

**Affiliations:** 1 Medicine, Imam Abdulrahman Bin Faisal University, Dammam, SAU; 2 Emergency Department, Imam Abdulrahman Bin Faisal University, Dammam, SAU; 3 Emergency Department, Imam Abdulrahman Bin Faisal University, King Fahad University Hospital, Dammam, SAU

**Keywords:** acute generalized exanthematous pustulosis, hydroxychloroquine, covid-19, cutaneous manifestations of covid-19

## Abstract

There is a dearth of robust evidence regarding coronavirus disease 2019 (COVID-19)-related coetaneous manifestations, complications and adverse treatment events. Upon review of the literature there are only a few cases reported of acute generalized exanthematous pustulosis (AGEP) in COVID‐19 patients after treatment.

Therefore, we are reporting a case of a 34-year-old male not known to have any chronic illness. His severe COVID-19 infection resolved four days prior to presentation to the Emergency Department with pustular rash on erythematous base over his face, neck, upper limbs, anterior and posterior trunk including oral cavity and tounge. The rash started after he took azithromycin, oseltamivir, ribavirin, lopinavir, hydroxychloroquine, prednisolone, ceftriaxone, clindamycin, interferon (IFN) beta, and ceftazidime for COVID-19. Skin punch biopsy was done and he was diagnosed with AGEP but it was still not known if it was related to COVID-19 or a drug-induced condition. Patient was treated with betamethasone valerate 0.1% ointment and lotion, promethazine hydrochloride 25mg tablet, paracetamol 500mg tablet, calcipotriol 50mcg/g and betamethasone 0.5mg/g gel. He discharged the same day to manage at home despite not improving.

In the end, we found only a few studies that describe the cutaneous manifestations of COVID-19 infection, which were mainly case reports. We can’t be sure that AGEP is a late and severe complication of COVID-19 infection. However, AGEP could be a rare adverse effect of hydroxychloroquine therapy. Improving the knowledge about a wide range of different signs and symptoms of the disease and its severity in addition to all possible adverse treatment events and complications can improve patient safety, survival rate, and quality of life.

## Introduction

Although cutaneous manifestations in coronavirus disease 2019 (COVID-19) patients are considered an uncommon presentation, there are various cutaneous manifestations reported including exanthems, purpura, urticaria, and varicella‐like vesicles. The first case was reported in Thailand where the patient presented with petechiae rash mimicking a dengue fever and then a few other cases followed [1]. Upon review of the literature we only found a few cases reported worldwide of acute generalized exanthematous pustulosis in COVID‐19 patients after treatment as in our case. The mechanism of this skin manifestation is still poorly understood and there is no evidence yet if it is related to the infection itself or is a serious side effect related to hydroxychloroquine which became widely utilized in the COVID‐19 pandemic. It is important to know these skin manifestations to help the clinicians in diagnosing patients who present with rare COVID-19 symptoms.

## Case presentation

**Figure 1:**
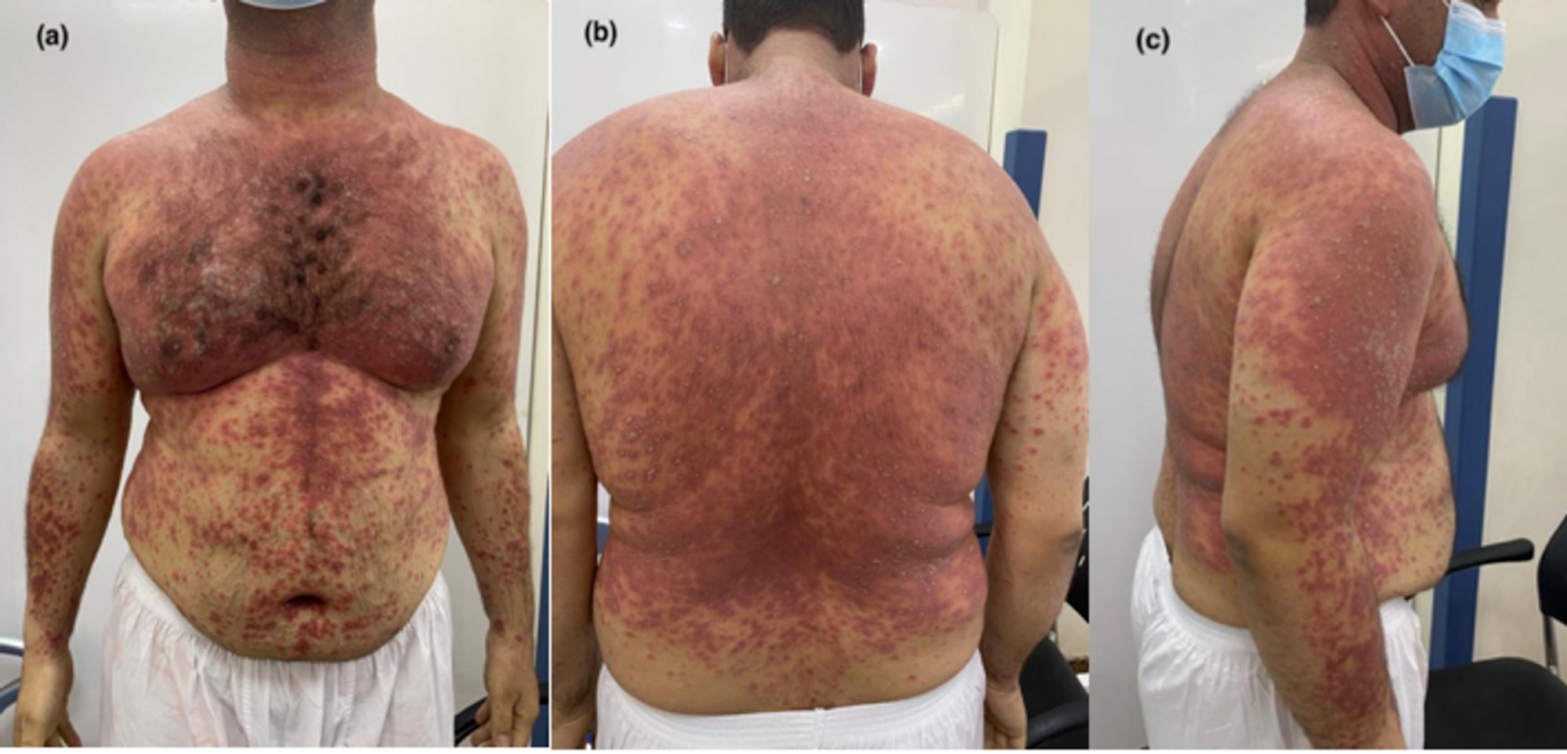
Clinical presentation of Acute Generalized Exanthematous Pustulosis a, b, c Marked erythema involved neck, upper limbs, anterior and posterior trunk with multiple distributed pustules.

A 34-year-old male not known to have any chronic illness resolved of severe COVID-19 infection four days before presenting to the Emergency Department with pustular rash on erythematous base over his face, neck, upper limbs, anterior and posterior trunk including oral cavity and tongue (Figure 1). He had no previous similar episode nor a family history of any dermatological disease and was not known to be allergic. He was admitted during the last three weeks for COVID-19 and treated with azithromycin, oseltamivir, ribavirin, lopinavir, hydroxychloroquine, prednisolone, ceftriaxone, clindamycin, interferon (IFN) beta and ceftazidime. Vital signs in the Emergency Department were temperature: 39 C, heart rate: 125 bpm, respiratory rate: 22 bpm, blood pressure: 122/60 mmHg, O2: 97% on room air. For the investigation, complete blood count (CBC) result was significant for white blood cells (WBCs) (Table 1).

**Table 1:** Blood investigations upon admission CBC: complete blood count, BUN: blood urea nitrogen, LDH: Lactate dehydrogenase, PT: prothrombin time, PTT: partial thromboplastin time, INR: international normalized ratio.

Investigations	Reference Range	Patient Result
CBC		
Total white blood cells	4.0-11.0 k/µL	16.2
Hemoglobin	13.0-18.0 g/dL	14.0
Biochemistry		
Creatinine	0.6-1.3 mg/dL	0.79
BUN	8.4-21 mg/dL	7
Ca+	8.4-10.2 mg/dL	8.1
Na+	136-146 mEq/L	137
K+	3.5-5.1 mEq/L	4.1
CL-	98-107 mEq/L	107
LDH	81-234 units/L	203
Total serum bilirubin	6.4-8.3 g/dL	5.9
Direct bilirubin	0.1-0.5 mg/dL	0.4
Albumin	3.2-5.2 g/dL	3.3
Alkaline phosphatase	40-150 units/L	76
Arterial Blood gases		
pH	7.35-7.45	7.45
pO_2_	83-108 mmHg	56.3
pCO_2_	35-45 mmHg	33.1
Coagulation profile		
PT	12.9-15.9 seconds	14.5
PTT	25.6-42.3 seconds	35.9
INR	-	1.05

Skin punch biopsy was done, and he was diagnosed with acute generalized exanthematous pustulosis (AGEP) but it was still not known if it was related to COVID-19 infection or a drug-induced condition. Patient treated with betamethasone valerate 0.1% ointment and lotion, promethazine hydrochloride 25mg tablet, paracetamol 500mg tablet, calcipotriol 50mcg/g and betamethasone 0.5mg/g gel. He was discharged the same day to manage at home despite not improving because the hospital was occupied with COVID-19 patients.

## Discussion

A study conducted by Suchonwanit et al. found that the majority of reported COVID-19 cases with cutaneous manifestations from January 1, 2020 to April 19, 2020 have diffuse/scattered papulovesicular lesions on the trunk followed by erythematous rash. Also, AGEP was documented as rare adverse effect of hydroxychloroquine and lopinavir/ritonavir treatment [2]. Similarly, a review done by Marraha et al. about dermatological manifestations of COVID-19 infection found that the most skin rashes were acral areas of erythema with vesicles or pustules and pernio-like lesions followed by erythematous maculopapular lesions [3].

Moreover, Litaiem et al. reported a case in Tunisia of a 39‐year‐old female patient diagnosed with COVID‐19 infection and 18 days after hydroxychloroquine (600 mg daily) initiation she developed cephalocaudal spread of erythematous and pustular plaques. The diagnosis of AGEP was made based on clinical and histopathological examinations. Eventually, the skin lesions were significantly improving after hydroxychloroquine withdrawal. The relation of AGEP and severe acute respiratory syndrome coronavirus 2 (SARS‐CoV‐2) infection or its treatment remain poorly understood [4]. The AGEP generally occurs within 48 hours of treatment initiation but, AGEP arising after hydroxychloroquine treatment is distinguishable by longer incubation period it can be up to two to three weeks. In our case the patient was started on 200 mg tablet orally every 12 hours of hydroxychloroquine treatment on June 5, 2020 after he was diagnosed with COVID-19 and he started to have this skin manifestation on June 27, 2020 while he still on treatment.

On the other hand, Galván Casas et al. conducted a prospective trial involving 375 cases of COVID-19 patients to study the cutaneous manifestations of COVID-19. He found that the most common skin lesions were maculopapular eruptions (47%) followed by areas of erythema with vesicles or pustules (19%) and urticarial lesions (19%). In addition to that, 59% of erythematous patterns associated with vesicles or pustules that tended to appear late in the evolution of the COVID-19 disease, as opposed to other skin manifestations which tend to appear in the presence of other COVID-19 symptoms [5].

One of the literature reviews reported that skin manifestations often occurred after initiation or withdrawal of the offending agent. The most known conditions were Johnson syndrome and Toxic Epidermal Necrolysis and the lesser-known AGEP [6]. However, most of those patients were non-COVID-19 patients and used hydroxychloroquine treatment for other reasons including rheumatological diseases. Moreover, the overlap between different cases makes it difficult to know whether these specific dermatological conditions are related to infection or drug-induced.

## Conclusions

In the end, we found only a few studies describing cutaneous manifestations of COVID-19 infection and the majority were case reports. We can’t confirm that AGEP be considered as a late and severe complication of COVID-19 infection. However, AGEP could be a rare adverse effect of hydroxychloroquine therapy. We still need more studies with a large sample size to be more accurate. Improving the knowledge about a wide range of different signs, symptoms of the disease and its severity in addition to all possible treatment adverse events and complications can improve patient safety, survival rate, and quality of life.
